# Multidisciplinary cooperative first aid model for the treatment of patients with pelvic and multiple fractures

**DOI:** 10.12669/pjms.38.3.5014

**Published:** 2022

**Authors:** Yun Han, Ganggang Peng, Lijun Liu, Xiaohua Xie

**Affiliations:** 1 Yun Han, Department of Traumatic Orthopedics, Shenzhen Second People’s Hospital, 3002 Sungang West Road, Shenzhen, 518030 Guangdong Province P.R. China; 2 Ganggang Peng, Department of Emergency, Shenzhen Second People’s Hospital, 3002 Sungang West Road, Shenzhen, 518030 Guangdong Province P.R. China; 3 Lijun Liu, Department of Traumatic Orthopedic, Shenzhen Second People’s Hospital, 3002 Sungang West Road, Shenzhen, 518030 Guangdong Province P.R. China; 4 Xiaohua Xie, Department of Nursing, Shenzhen Second People’s Hospital, 3002 Sungang West Road, Shenzhen, 518030 Guangdong Province P.R. China

**Keywords:** Multidisciplinary cooperative first aid model, Emergency system, Pelvic fracture, Multiple fractures, Treatment

## Abstract

**Objectives:**

To retrospectively evaluate a multidisciplinary cooperative first aid model for the treatment of patients with pelvic and multiple fractures in the emergency department.

**Methods:**

The records of patients with pelvic fractures complicated with multiple fractures treated in our hospital from February 2020 to April 2021 were selected, of which 34 patients received conventional trauma first aid mode control group) and 34 patients received multidisciplinary joint first aid mode (study group). We compared pelvic function (Majeed functional score) and fracture reduction outcomes, as well as serum inflammatory factor levels and complication rates after treatment between the two groups.

**Results:**

The Majeed score in the study group (90. 15 ± 6.83) was higher than that in the control group (75. 47 ± 5.35), and the differences were statistically significant(P<0.05). The value for combined excellent and good rates of fracture reduction in the study group (85.29%, 29/34) was significantly higher than that in the control group (58.82%, 20/34), and the difference was statistically significant(P<0.05). We found similar levels of TNF-a and IL-6 between the two groups at admission(P>0.05); however, the serum levels of TNF-a and IL-6 in the study group were lower than those in the control group on the fifth day after admission, and the difference was statistically significant (P<0.05 or P<0.01). The incidence of complications in the study group (17.64%, 6/34) was significantly lower than that in the control group (61.76%, 21/34), the difference was statistically significant(P<0.05).

**Conclusion:**

The multidisciplinary cooperative first aid model for the treatment of patients with pelvic and multiple fractures can effectively shorten the treatment time, increase the excellent functional rehabilitation rate, inhibit the release of inflammatory factors, and reduce the incidence of complications (such as infections), when compared to the conventional trauma emergency system.

## INTRODUCTION

Economic, transportation, and construction industries are rapidly developing in many countries; in addition to natural disasters, common industrial and traffic accidents require the attention of trauma and public emergency teams. Patients requiring emergency medical treatment and critically ill patients have increased in numbers. Severe trauma patients often need immediate life-saving treatments.^1^,^2^ Surgical treatments need to be prioritized for patients with life-threatening injuries.^3^,^4^ For many patients with serious organ injury and poor general condition, timely hemostasis and control technologies are used initially to stabilize the patient’s condition before attempting a reparative operation.

Patients with pelvic and other multiple fractures need to be admitted promptly to an intensive care unit to improve the success rate of treatment and prevent complications such as multiple organ failure or respiratory tract and urinary tract infections.^5^ The first aid of pelvic fracture combined with multiple fractures is very important. Improper treatment can aggravate the injury, increase the patient’s pain, and even form disability and endanger life. Therefore, it is very important to carry out reasonable and effective first aid in time. Trauma refers to the destruction or dysfunction of the integrity of tissue structure caused by mechanical injury factors on the human body. The type of trauma is determined according to the injury factors, injury location, skin integrity and the severity of the injury. Severe trauma can cause systemic reaction, and local manifestations include pain, swelling and tenderness in the injury area; There are deformities and dysfunction in fracture and dislocation, which may also lead to fatal bleeding, shock, asphyxia and disturbance of consciousness.^6^multidisciplinary cooperative first aid models can shorten the pre-admission treatment time, increase the hospital emergency team success rates, coordinate treatments from different medical specialties, and effectively manage time. For this retrospective study, we compared fracture repair outcomes between two groups of patients with pelvic and multiple fractures who received treatment by either the conventional or a multidisciplinary cooperative first aid model.

## METHODS

The records of patients with pelvic fractures complicated with multiple fractures treated in our hospital from February 2020 to April 2021 were selected, of which 34 patients received conventional trauma first aid mode and 34 patients received multidisciplinary cooperative first aid model. There were 20 men and 14 women in the study group, their mean was 37.2 ± 4.6 years. The causes of trauma included crush injury (4 cases), falls (8 cases), and traffic accidents (22 cases). The average interval between the incident and the medical service was 4.2 ± 1.8 hours (1 to 7 h). There were 19 men and 15 women in the control group (15 patients with type B and 19 with type C pelvic fractures). Their mean age was 36.8 ± 5.2 years. The injury causes included five crush injuries, nine falls, and 20 traffic accidents. The average interval between the incident and the medical service was 4.1 ± 1.9 hours (2 to 7 h). We found no significant general information differences between the two groups(P>0.05). The Medical Ethics Committee of our hospital approved this study (Ethical approval number (No. 2021022, Data: 2021 April 7th).

### Inclusion criteria:

- Patients older than 18 years.
- Patients with imaging examination consistent with pelvic and multiple fractures.
-Patients choosing to participate and sign an informed consent.

### Exclusion criteria:

-Patients with pathological fractures.
- Patients with non-traumatic fractures.
- Patients with coagulation dysfunction or severe organ diseases before admission.
- Patients with mental diseases.

In the conventional trauma first aid mode, team goes to the scene immediately after receiving the call for help to evaluate the patient’s injury, opens the venous pathway for infusion and rescue shock, quickly and accurately bind the wound with nylon net cover, gauze bandage or other available cloth to stop bleeding. Pelvic band or cloth strip is used to bind around the pelvis. After admission, the patient’s pain is controlled. At the same time, oxygen inhalation, hemostasis and correction of water electrolyte balance are carried out. Emergency orthopaedic surgery is actively carried out according to the patient’s trauma type and fracture site.

In the multidisciplinary cooperative first aid model, treatment is carried out by professional surgeons, radiologists and corresponding supporting facilities (CT, fluorescence examination equipment, operating room, etc.) in the conventional trauma first aid mode. Preliminarily judgement of the severity of trauma in performed in the ambulance. In case of pelvic fracture with unstable hemodynamics, patients are transferred to the hospital for CT examination and CT arteriography immediately. Vascular embolization is implemented in case of arterial bleeding. In the process of pre-hospital first aid and transportation, the different methods of existing information communication (such as WeChat group and QQ group) are used between pre-hospital and in-hospital first aid personnel to implement an effective early warning classification system and to improve the timeliness of emergency treatment. Patient’s comprehensive medical history, physical examination and vital signs are reassessed after admission. Fast and effective infusion channel are established and the infusion scheme is adopted based on crystal solution and concentrated red blood cells. Temporary fixation of fracture with external stent or plaster is done. VSD covered negative pressure drainage is performed in patients with large skin defect. If the patient’s vital signs are stable, the corresponding multidisciplinary team (trauma orthopedics, general surgery, thoracic surgery, urology and obstetrics and Gynecology) can immediately carry out surgical treatment (early rectostomy, cystostomy, vaginal revision, etc. according to the specific injury situation, to reduce the incidence of infection complications. If the patient’s vital signs are unstable, after being transferred to ICU, coagulation dysfunction correction is immediately performed. Hypothermia, and acidosis are treated. Anti-infection intervention is carried out, and vital signs are constantly monitored to find abnormalities in time and avoid serious complications. After stabilization of vital signs, corresponding multidisciplinary team cooperation was given for surgical treatment.

**Table-I:** Comparison of excellent and good rate of fracture reduction between the two groups 4 weeks after operation[n(%)].

Group	n	perfect	Good	Acceptable,	Poor	Excellent rate
Study	34	14(41.18)	15(44.11)	4(11.76)	1(2.94)	29(85.29)
Control	34	7(20.59)	13(38.23)	9(26.47)	5(14.71)	20(58.82)
*χ^2^*						12.936
*P*						0.000

### Follow-up

One year after operation, the pelvic function was evaluated by Majeed function score, with a score of 0~100. The lower the score, the worse the pelvic function. Fracture reduction assessment at four weeks after operation was as follows: the postoperative fracture displacement is considered better if it is less than 4mm; 4~10mm is good, 11~20mm is acceptable, and>20mm is poor.

Postoperative pelvic CT plain scan was used to determine the reduction of pelvic fracture. The clinical judgment criteria of fracture reduction were as follows: maximal residual displacement after treatment of 0~5.0 mm, 6.0~10.0 mm, 11.0~15.0 mm and more than 15.0 mm. Levels of serum inflammatory factors TNF-a and IL-6 and the incidence of complications were assessed before the admission and 5 days after operation.

Statistical analysis of the data was performed using SPSS 26.0 software. We measured and compared *t*-test values between groups. We used chi square tests to compare count data results (n, percentages). We considered any P < 0.05 as statistically significant.

All procedures performed in studies involving human participants were in accordance with the ethical standards of the ethics committee of the Shenzhen Second People’s Hospital (No. 2021022, Data: 2021 April 7th).

## RESULTS

The optimal and good fracture reduction rate(as indicated by pelvic CT plain scan results) in the study group (85.29%, 29/34) was significantly higher than that in the control group (58.82%, 20/34) with a statistically significant difference (P < 0.05).The Majeed score in the study group (90.15 ± 6.83) was higher than that in the control group (75.47 ± 5.35) with a statistically significant difference (P < 0.05).

We found similar levels of TNF-a and IL-6 between the two groups at admission(P>0.05). The serum levels of TNF-a and IL-6 in the study group were lower than those in the control group on the fifth day after admission, and the differences were statistically significant (P < 0.05 or P < 0.01).

The incidence of complications in the study group (17.64%; 6/34) was significantly lower than that in the control group (61.76%; 21/34) with a statistically significant difference (P < 0.05).

**Table-II:** Comparison of Majeed score between the two groups one year after operation (*x*¯±s).

Group	n	Majeed score
Study Group	34	90.15±6.83
Control Group	34	75.47±5.35
*t*		2.483
*P*		0.005

**Table-III:** Comparison of serum inflammatory factors between the two groups before admission and 5 days after operation(n = 34;*x*¯±s).

Group	TNF-a(g/L)		IL-6(ng/L)	
	
	On admission	Admission day 5	On admission	Admission day 5
Study	84.96 ± 5.47	39.32 ± 2.04	25.07 ± 3.46	11.09 ± 1.21
Control	83.68 ± 5.09	65.82 ± 4.58	24.85 ± 3.27	17.63 ± 2.28
*t*	15.043	3.452	13.916	4.085
*P*	0.847	0.000	0.734	0.000

**Table-IV:** Comparison of 1-year postoperative complication rate between the two groups [n(%)].

Group	n	Deep Infection	Fracture deformity	Multiple organ failure	Other	Totally
Study	34	1(2.94)	2(5.88)	1(2.94)	2(5.88)	6(17.64)
Control	34	6(17.65)	8(23.53)	3(8.82)	4(11.76)	21(61.76)
*χ^2^*						10.384
*P*						0.000

## DISCUSSION

The results showed that the Majeed score of patients with pelvic fracture combined with multiple fractures in multidisciplinary assisted first aid mode (90.15±6.83) was higher than that in conventional first aid mode (75.47±5.35), and the excellent and good rate of fracture reduction in multidisciplinary assisted first aid mode was 85.29%, significantly higher than 58.82% in conventional first aid mode. Our results show that pelvic function and fracture reduction effect in patients is good after the multidisciplinary assistance first aid mode is adopted. Comparison of TNF-a and IL-6 levels between the two first aid modes showed that on the 5th day after admission, the levels of serum TNF-a and IL-6 in the multidisciplinary assisted first aid mode were lower than those in the conventional first aid mode. These results suggest that the multidisciplinary assisted first aid mode can more effectively inhibit the release of inflammatory factors. The incidence of complications in multidisciplinary assisted first aid mode was 17.64%, which was significantly lower than 61.76% in conventional first aid mode. Bouman AI et al.^11^ conducted a prospective, multicenter, non-randomized controlled study of 132 cases of multiple fractures. In this study, 65 patients received a comprehensive and coordinated treatment mode that included trauma surgeons and rehabilitation physicians. The results show that the multidisciplinary cooperation mode can more effectively improve the functional status and quality of life of patients with multiple injuries than the normal scale mode. Fan H et al.^12^ described control experiment assessing application effect of injury control orthopedic emergency (DCO) mode (treatment according to the patient’s physiological tolerance stage, simplifying the initial operation, then resuscitation in ICU, and finally final operation in patients with pelvic fractures complicated with multiple fractures. Six months after treatment, 47 patients that received DCO mode had significantly higher physiological function (PF), scores of physical pains (BP), role physiology (RP), emotional function (EF), social function (SF), vitality and general health (GH) and mental health than those in the control group (P<0.05). The above results are consistent with the results of this study.

In the current study, we compared outcomes of fracture repair in pelvic fracture patients treated by conventional or a coordinated integrated emergency system. Advanced trauma life support (ATLS)^13^ is a standard process and specification of disaster management and emergency preparedness that was adopted by the American College of Surgeons Committee on Trauma in 1979.

ATLS protocols, when initiated at a trauma center, can improve the performance of the previously described procedures and may improve short-term mortality as well. ATLS describes plans for various disaster states in advance to improve the response ability in case of events through regular training and drills, and minimize the consequences of disasters. It addresses advantages and disadvantages in the plan, personnel training, materials, site, etc. in case of major disaster events. Specific arrangements are made for the comprehensive clinical treatment of patients with multiple and compound injuries and the construction of a regional severe trauma treatment system.^14^ Unlike interdisciplinary and transdisciplinary collaborations, a multi-disciplinary assistance first aid model, described in the current study, relies on a combination of different expertise and employs a professional team composed of multiple disciplines and specialties. The quality of team members is relatively high, which requires not only excellent professional skills, but also high emergency response ability and physical quality.^15^ Nurses should have unique emergency consciousness and keen thinking, and the observation of the condition should be comprehensive and predictable.^16^ During the observation, it is necessary to understand the patient’s injury mode, injury tools, injury position and focus from the patient’s family and the perpetrator, and conduct a comprehensive analysis. It also requires collecting extensive information from the patient and the patient’s family to make a comparison with the patient’s pre-injury state, fully eliminate the interference of other factors on the observation indicators, make a correct injury assessment and improve the success rate of the rescue. 17

### Limitations of the study

First of all, we did not assess the details of the treatment of pelvic and multiple fractures. Second, there were no records available of patients’ satisfaction with the pelvic and multiple fractures treatment. Finally, this was a retrospective study, and the number of cases was too small to assume a lack of confounding factors. Future prospective studies with larger cohorts are needed to confirm these results.

## CONCLUSIONS

This study shows that the integral coordinated trauma emergency system for the emergency treatment of pelvic and multiple fractures can effectively shorten the treatment time, improve the optimal functional rehabilitation rate, inhibit the release of inflammatory factors, reduce the incidence of complications such as infection, and can be popularized for clinical application.

### Authors’ Contributions:

**YH:** Designed the project. **GP, XW, MH & LL:** Were involved in data collection and data analysis. **YH:** Prepared the manuscript. **XX:** Edited the manuscript, made significant contribution to the study and is also responsible and accountable for the accuracy or integrity of the work. All authors read and approved the final manuscript.

## References

[1] Callejas-Moraga EL, Casado E, Gomez-Nuñez M, Caresia-Aroztegui AP (2020). Severe osteomalacia with multiple insufficiency fractures secondary to intravenous iron therapy in a patient with Rendu-Osler-Weber syndrome. Bone Rep.

[2] Wu C, Hong Y, Wu B, Huang B (2021). The Effects of Add-On Self-Care Therapy on Epidural Catheter Analgesia and Pain in Patients after Surgical Stabilization of Multiple Rib Fractures. Pain Manag Nurs Off J Am Soc Pain Manag Nurses. Pain Manag Nurs.

[3] Belaya Z, Golounina O, Nikitin A, Tarbaeva N, Pigarova E, Mamedova E (2021). Multiple bilateral hip fractures in a patient with dyskeratosis congenita caused by a novel mutation in the PARN gene. Osteoporos Int J Establ Result Coop Eur Found Osteoporos Natl Osteoporos Found USA.

[4] Duan K, Li Y, Yang W (2021). Discrete element method simulation of the growth and efficiency of multiple hydraulic fractures simultaneously-induced from two horizontal wells. Geomech Geophys Geo-Energy Geo-Resour.

[5] Zhang H, Zhang Y, Wu J, Li Y, Zhou X, Li X (2020). Risks and features of secondary infections in severe and critical ill COVID-19 patients. Emerg Microbes Infect.

[6] Liu Z, Tang G, Guo S, Cai B, Li Q (2020). Therapeutic effects of internal fixation with support plates and cannulated screws via the posterolateral approach on supination external rotation stage IV ankle fracture. Pak J Med Sci.

[7] Chen HT, Wang YC, Hsieh CC (2019). Trends and predictors of mortality in unstable pelvic ring fracture:a 10-year experience with a multidisciplinary institutional protocol. World J Emerg Surg.

[8] Uludag N, Tötterman A, Beckman MO, Sundin A (2018). Anatomic distribution of hematoma following pelvic fracture. Br J Radiol.

[9] Skitch S, Engels PT (2018). Acute Management of the Traumatically Injured Pelvis. Emerg Med Clin North Am.

[10] Basat NB, Allon R, Nagmi A, Wollstein R (2017). Treatment of open fractures of the hand in the emergency department. Eur J Orthop Surg Traumatol.

[11] Bouman AI, Hemmen B, Evers SM (2017). Effects of an Integrated 'Fast Track'Rehabilitation Service for Multi-Trauma Patients:A Non-Randomized Clinical Trial in the Netherlands. PLoS One.

[12] Fan H, Fei R, Guo C, Li Y, Yan C, Chen F, Zhang Y (2021). Effects of emergency treatment mode of damage-control orthopedics in pelvic fracture complicated with multiple fractures. Am J Transl Res.

[13] Galvagno SM, Nahmias JT, Young DA (2019). Advanced Trauma Life Support®Update 2019:Management and Applications for Adults and Special Populations. Anesthesiol Clin.

[14] Farrell MS, Emery B, Caplan R, Getchell J, Cipolle M, Bradley KM (2020). Outcomes with advanced versus basic life support in blunt trauma. Am J Surg.

[15] Najm A, Kostine M, Pauling JD (2020). Multidisciplinary collaboration among young specialists:results of an international survey by the emerging EULAR network and other young organisations. RMD Open.

[16] Sari D, Baysal E, Celik GG, Eser I (2018). Ethical Decision Making Levels of Nursing Students. Pak J Med Sci.

[17] Ugur E, Demir H, Akbal E (2015). Postgraduate education needs of Nurses'who are caregivers for patients with diabetes. Pak J Med Sci.

